# Impaired hypoxic pulmonary vasoconstriction in a mouse model of Leigh syndrome

**DOI:** 10.1152/ajplung.00419.2018

**Published:** 2018-12-06

**Authors:** Grigorij Schleifer, Eizo Marutani, Michele Ferrari, Rohit Sharma, Owen Skinner, Olga Goldberger, Robert Matthew Henry Grange, Kathryn Peneyra, Rajeev Malhotra, Martin Wepler, Fumito Ichinose, Donald B. Bloch, Vamsi K. Mootha, Warren M. Zapol

**Affiliations:** ^1^Anesthesia Center for Critical Care Research of the Department of Anesthesia, Critical Care, and Pain Medicine, Harvard Medical School and Massachusetts General Hospital, Boston, Massachusetts; ^2^Howard Hughes Medical Institute and Department of Molecular Biology, Harvard Medical School and Massachusetts General Hospital, Boston, Massachusetts; ^3^Cardiology Division and Cardiovascular Research Center, Department of Medicine, Harvard Medical School and Massachusetts General Hospital, Boston, Massachusetts; ^4^Institut für Anästhesiologische Pathophysiologie und Verfahrensentwicklung, Ulm, Germany; ^5^Division of Rheumatology, Allergy and Immunology, Department of Medicine, Harvard Medical School and Massachusetts General Hospital, Boston, Massachusetts

**Keywords:** complex I, hypoxic pulmonary vasoconstriction, Leigh syndrome, mice, mitochondria

## Abstract

Hypoxic pulmonary vasoconstriction (HPV) is a physiological vasomotor response that maintains systemic oxygenation by matching perfusion to ventilation during alveolar hypoxia. Although mitochondria appear to play an essential role in HPV, the impact of mitochondrial dysfunction on HPV remains incompletely defined. Mice lacking the mitochondrial complex I (CI) subunit Ndufs4 (*Ndufs4*^−/−^) develop a fatal progressive encephalopathy and serve as a model for Leigh syndrome, the most common mitochondrial disease in children. Breathing normobaric 11% O_2_ prevents neurological disease and improves survival in *Ndufs4*^−/−^ mice. In this study, we found that either genetic *Ndufs4* deficiency or pharmacological inhibition of CI using piericidin A impaired the ability of left mainstem bronchus occlusion (LMBO) to induce HPV. In mice breathing air, the partial pressure of arterial oxygen during LMBO was lower in *Ndufs4*^−/−^ and in piericidin A-treated *Ndufs4*^+/+^ mice than in respective controls. Impairment of HPV in *Ndufs4*^−/−^ mice was not a result of nonspecific dysfunction of the pulmonary vascular contractile apparatus or pulmonary inflammation. In *Ndufs4*-deficient mice, 3 wk of breathing 11% O_2_ restored HPV in response to LMBO. When compared with *Ndufs4*^−/−^ mice breathing air, chronic hypoxia improved systemic oxygenation during LMBO. The results of this study show that, when breathing air, mice with a congenital Ndufs4 deficiency or chemically inhibited CI function have impaired HPV. Our study raises the possibility that patients with inborn errors of mitochondrial function may also have defects in HPV.

## INTRODUCTION

Hypoxic pulmonary vasoconstriction (HPV) is a physiological vasomotor response of the small pulmonary resistance arteries to alveolar hypoxia. HPV contributes to the maintenance of normal systemic oxygenation by redistributing blood flow away from poorly ventilated lung regions, thereby matching alveolar ventilation with perfusion (22, 31).

Mitochondrial complex I (CI) is a major component of the electron transport chain (ETC) and contributes both to the oxidation of NADH and to the generation of a proton gradient across the mitochondrial inner membrane to drive ATP production (37). Mutations in either nuclear or mitochondrial genes that encode proteins involved in the assembly and/or the electron transport function of CI cause mitochondrial dysfunction (33).

The signals that trigger HPV are incompletely understood. It is hypothesized that CI “senses” alveolar hypoxia and initiates the depolarization of pulmonary arterial smooth muscle cells, which results in calcium entry and subsequent vasoconstriction. Previous studies showed that pharmacological inhibition of CI impaired the ability of pulmonary smooth muscle cells to induce vasoconstriction in isolated lung models of HPV (2, 36). However, to our knowledge, no study has previously evaluated HPV in a genetic model of complex I deficiency in vivo.

CI deficiency, caused by a deletion in the nuclear encoded gene *Ndufs4*, results in Leigh syndrome, which is the most common mitochondrial disease in children (8, 30). To date, mutations in more than 75 different genes can cause Leigh syndrome, which is a devastating neurodegenerative disease leading to death typically within the first years of life (17). Mice lacking Ndufs4, a protein which is responsible for the assembly and normal function of CI, serve as a model of Leigh syndrome. *Ndufs4*^−/−^ mice (knockout mice) develop a progressive neurodegenerative disease and die ~60 days after birth (18). We previously reported that continuously breathing normobaric 11% O_2_ prevents neurological disease and improves survival in *Ndufs4*^−/−^ mice (14). The objective of this study was to investigate the effect of Ndufs4 deficiency and pharmacological inhibition of CI on HPV in vivo. We hypothesized that congenital deficiency and pharmacological inhibition of CI impair HPV and worsen arterial systemic oxygenation. To characterize the beneficial effects of chronic hypoxia in *Ndufs4* deficient mice better, we also investigated whether exposure to breathing 11% O_2_ for 3 wk would restore HPV and improve systemic oxygenation in *Ndufs4*^−/−^ mice.

## MATERIALS AND METHODS

### 

#### Animals.

All animal experiments outlined in this study were approved by the Subcommittee on Research Animal Care of the Massachusetts General Hospital. We studied Ndufs4-deficient (*Ndufs4*^−/−^) mice and their wild-type (*Ndufs4*^+/+^) littermates on a C57BL6/J genetic background of both sexes with an age range of 4–5 wk and a weight range between 12 and 17 g. *Ndufs4*^−/−^ mice were kindly provided by the Palmiter laboratory at the University of Washington. *Ndufs4*^−/−^ and their *Ndufs4*^+/+^ littermates were housed and bred in the hospital’s animal facility. Animals for each experiment were matched for sex and age.

#### Measurement of HPV in mice.

To measure HPV, left lung pulmonary vascular resistance index (LPVRI) was calculated before and during alveolar hypoxia. Surgical preparation was performed as described previously (4, 12, 39). Briefly, general anesthesia was induced by intraperitoneal injection of ketamine (120 mg/kg) and fentanyl (0.09 mg/kg). Mice were placed on a heating pad to maintain core temperature at 37°C. Following a tracheostomy, muscle relaxation was induced by intraperitoneal injection of rocuronium (1 mg/kg), and volume-controlled ventilation was initiated at a respiratory rate of 110 breaths/min and a tidal volume of 8 ml/kg. Inspired oxygen fraction of 1.0 and a positive end-expiratory pressure of 1 cm H_2_O were provided throughout the measurements using MiniVent 845 (Harvard Apparatus, Holliston, MA). A polyethylene catheter was placed in the right carotid artery and the right jugular vein for hemodynamic monitoring and infusion of 0.06 ml·h^−1^·g^−1^ lactated Ringer solution. After left parasternal thoracotomy, a precision perivascular flow probe (MA-0.5PSB, Transonic Systems, Ithaca, NY) was placed around the left pulmonary artery to measure pulmonary blood flow (Q_LPA_). A fluid-filled polyethylene 10 catheter was inserted into the main pulmonary artery for measurement of pulmonary arterial pressure (PAP). Q_LPA_, heart rate (HR), mean arterial pressure (MAP), and PAP were continuously measured and recorded with a digital data software (Chart 5, AD Instruments, Colorado Springs, CO). To calculate LPVRI, transient reduction of Q_LPA_ was carried out by three partial occlusions of the inferior vena cava as described previously (13). LPVRI was calculated from the slopes of the regression line representing the PAP/Q_LPA_ relationship. HPV was expressed as the percentage increase in LPVRI during left mainstem bronchial occlusion (LMBO)-induced alveolar hypoxia from baseline LPVRI and was calculated as (LPVRI_duringLMBO_ − LPVRI_beforeLMBO_)/LPVRI_beforeLMBO_·100 (21). To assess systemic oxygenation and estimate intrapulmonary shunt fraction, arterial and mixed venous blood gas analysis was performed using the ABL800 FLEX analyzer (Radiometer America, Westlake, OH). Blood samples (80 μl) were obtained simultaneously from the right carotid artery and common pulmonary artery after hemodynamic measurements.

#### Measurement of intrapulmonary shunt fraction.

Intrapulmonary shunt (Qs/Qt) estimates the fraction of the total cardiac output that does not take part in gas exchange and passes unoxygenated into arterial circulation (6, 18). Qs/Qt was calculated in *Ndufs4*^−/−^ and *Ndufs4*^+/+^ mice breathing either air or 11% O_2_. Blood samples were simultaneously obtained from the carotid and main pulmonary arteries and analyzed for arterial ($\text{P}{\text{a}}_{\text{O}}{2_{}}_{}$) and mixed-venous oxygen partial pressure. Oxygen content of arterial, mixed venous, and pulmonary end-capillary blood was calculated assuming a hemoglobin oxygen-binding capacity of 1.34 ml/g. Saturation of pulmonary end-capillary blood oxygen was assumed to be 1.0. Qs/Qt was calculated with the Berggren equation (5).

#### Measurement of lower thoracic aortic flow.

Cardiac output was estimated by measuring lower thoracic aortic flow (Q_LTAF_) before and during LMBO using the MA-0.5PSB flow probe connected to a flowmeter (TS-420 module, Transonic Systems, Ithaca, NY). To estimate total systemic vascular resistance index (TSVRI), Q_LTAF_ was transiently reduced three times by partial occlusions of the inferior vena cava as described previously (12, 38). TSVRI was calculated from the slopes of the regression line representing the MAP/Q_LTAF_ relationship.

#### Administration of piericidin A or angiotensin II to mice.

Piericidin A, an NADH/ubiquinone oxidoreductase complex I inhibitor, was purchased from VWR International (Radnor, PA). Piericidin A dissolved in 100% DMSO, or DMSO alone (vehicle) was administered intraperitoneally in *Ndufs4*^+/+^ mice 30 min before HPV measurement. The amount of DMSO used in these studies did not exceed 1 ml/kg. Angiotensin II, a potent vasoconstrictor, was obtained from VWR International and dissolved in saline solution. A programmable syringe pump (BS-300, Braintree Scientific, Braintree, MA) was used to continuously infuse 0.5 µg·kg^−1^·min^−1^ angiotensin II via right jugular vein. Before and during infusion of angiotensin II, LPVRI was measured as described above. The dose and timing of angiotensin II administration was selected based on a previous study (9).

#### Mice breathing 11% O_2_.

Thirty-five-day-old mice were exposed to either air or 11% hypoxia and housed in 80-liter transparent acrylic boxes for 21 days. A membrane technology nitrogen generator (MAG-20, Higher Peak) was used to adjust relative concentration of nitrogen and oxygen in the chamber to obtain a continuous inspired oxygen concentration of 11% or air. The oxygen concentration was monitored daily using an O_2_ sensor (MiniOx 1, OhioMedical). CO_2_ levels were continuously monitored inside the chambers (CO200, Extech), and the CO_2_ concentration was maintained below 0.4% by adjusting the gas flow between 5 and 10 l/min. Soda lime was used as a CO_2_ scavenger inside the chambers. Mice were housed in cages with standard bedding, and a 12-h:12-h light-dark cycle was used throughout the experiment.

#### Measurement of succinate, fumarate, and cytokine mRNA levels in lung tissue.

To characterize the function of complex II in mice with deficiency of the *Ndufs4* gene better, we measured the levels of succinate and fumarate in lung tissues of *Ndufs4*^−/−^ and *Ndufs4*^+/+^ mice treated either with air or 11% O_2_. Following HPV measurements, the ventilated lungs were excised and snap-frozen in liquid nitrogen. Liquid chromatography mass spectrometry was used to measure metabolites in the lungs. Metabolites were extracted from lung samples on dry ice with 1.5 ml of 80% methanol. The tissue samples were homogenized with a Qiagen TissueLyser for 4 min. Samples were then centrifuged at 21,100 *g* for 20 min, and 750 µl of supernatant was collected, diluted to 30% in methanol, and lyophilized overnight. The lyophilized powder was resuspended in 70% acetonitrile. Liquid chromatography mass spectrometry measurements were performed on a Q-Exactive Plus Orbitrap mass spectrometer coupled with a Dionex UltiMate 3000 ultra-high performance liquid chromatography system (Thermo Fisher Scientific, Waltham, MA). Metabolites were separated on an Xbridge amide HILIC column (2.1 × 100 mm, 2.5 μM particle size; Waters, 186006091). Mobile phase A was 20 mM ammonium acetate and 0.25% ammonium hydroxide (pH 9) with 5% acetonitrile as described previously (15). Mobile phase B was 100% acetonitrile. Data acquisition was performed in full scan mode, with a range of 70–1,000 mass-to-charge ratio, resolving power of 140,000 (at 200 mass-to-charge ratio), an automatic gain control target of 3e6, and a maximum injection time of 400 ms.

To analyze the inflammatory response in the lung tissue of mice with the *Ndufs4* deficiency, we measured cytokine levels in lung tissue obtained from *Ndufs4*^−/−^ and *Ndufs4*^+/+^ mice breathing air. Real-time qPCR was used to measure the levels of IL-6, TNFα, and IL-1β mRNAs as described previously (3). Briefly, total RNA was extracted by the phenol/guanidinium method and reverse transcription was performed using MultiScribe MuLV Reverse Transcriptase (Thermo Fisher Scientific). A Mastercycler ep realplex (Eppendorf, Hamburg, Germany) was used for real-time amplification and quantification of mRNA transcripts. All mRNA levels were determined by the relative CT method normalized to 18S ribosomal RNA. TaqMan gene expression assays were used to quantify mRNA levels encoding IL-6, TNFα, and IL-1β, as well as the level of 18S RNA.

#### Measurement of NADH/NAD^+^ ratio in lung tissues.

Total NAD (NADH and NAD^+^) and NADH were measured to determine the NADH/NAD^+^ ratio in lung tissue using a NAD/NADH quantitation colorimetric kit (K337-100, BioVision, Inc.) in accordance with manufacturer’s protocol (24). Lung tissue was homogenized in extraction buffer and centrifuged at 14,000 *g* for 5 min. The amount of NAD and NADH was measured in the supernatant, and NAD^+^ was calculated by subtracting NADH from total NAD.

#### Statistical analysis.

Statistical analyses were performed using Prism 7 (GraphPad Software, La Jolla, CA) and RStudio Version 1.0.136 (RStudio, Boston, MA). The Shapiro-Wilk test was used to determine the normality of data variables. Multiple group differences in normally distributed data were determined with the one-way ANOVA with a post hoc Bonferroni correction. A Kruskal-Wallis test with post hoc Dunn’s comparison testing was used for data that were not normally distributed. Within the same experimental group, measurements were compared with the paired or unpaired Student’s *t*-test or Mann-Whitney test, as appropriate. Adjusted *P* values < 0.05 were considered statistically significant. All data are expressed as means ± SE or medians with interquartile ranges, as appropriate.

## RESULTS

### 

#### Effect of Ndufs4 deficiency or pharmacological inhibition of CI on HPV.

**Fig. 1.**
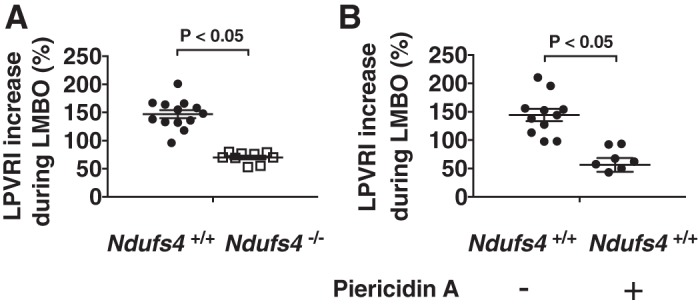
Hypoxic pulmonary vasoconstriction in Ndufs4*-*deficient mice. The percentage increase in LPVRI was lower in *Ndufs4*^−/−^ mice [*n* = 6 (male), *n* = 3 (female)] compared with *Ndufs4^+/+^* mice [*n* = 9 (male), *n* = 4 (female), *Ndufs4*^−/−^ vs. *Ndufs4^+/+^*, *P* < 0.05] (*A*). Treatment with the complex I inhibitor piericidin A (0.5 mg/kg) 30 min before HPV measurements impaired HPV in *Ndufs4^+/+^* mice [*n* = 4 (male), *n* = 3 (female)] compared with control *Ndufs4^+/+^* mice [*n* = 7 (male), *n* = 4 (female), piericidin A-treated *Ndufs4^+/+^* vs. DMSO-treated *Ndufs4^+/+^*, *P* < 0.05] (*B*). LPVRI was calculated from the slope of the PAP/Q_LPA_ relationship at baseline and during LMBO. HPV is denoted as the percentage increase in LPVRI induced by LMBO. Group differences were compared by Student’s *t*-test. Data are expressed as means ± SE. HPV, hypoxic pulmonary vasoconstriction; LMBO, left mainstem bronchial occlusion; LPVRI, left pulmonary vascular resistance index; PAP, pulmonary arterial pressure; Q_LPA_, left pulmonary arterial blood flow.

To investigate the effect of Ndufs4 deficiency on HPV, the LPVRI was measured at baseline and during LMBO in *Ndufs4*^−/−^ and *Ndufs4*^+/+^ mice. At baseline (before LMBO), with mice breathing 100% oxygen, hemodynamic parameters and LPVRI did not differ between the two genotypes (Table 1). In *Ndufs4*^−/−^ mice, LMBO induced a smaller increase in LPVRI, compared with *Ndufs4*^+/+^ mice (%increase in LPVRI in *Ndufs4*^−/−^ vs. *Ndufs4*^+/+^ mice: 70 ± 3 vs. 147 ± 7%, *P* < 0.05, Fig. 1*A*). During LMBO, there was no difference in hemodynamic parameters, including HR, MAP, and PAP between the two genotypes (Table 1). To investigate whether changes in systemic vascular resistance might explain the differences in HPV between wildtype and *Ndufs4*^−/−^ mice, the Q_LTAF_ and MAP were measured before and during LMBO. Q_LTAF_ and MAP did not differ between *Ndufs4*^−/−^ and *Ndufs4*^+/+^ mice at baseline or during LMBO (Table 2). The TSVRI, obtained from the relationship between Q_LTAF_ and MAP, was also similar at baseline between the two genotypes and was not altered by LMBO (Table 2). Taken together, these results show that the absence of CI subunit Ndufs4, and the resulting decreased CI activity, leads to impaired HPV but does not alter systemic vascular resistance either at baseline or during LMBO.

**Table 1.** Hemodynamic measurements before (baseline) and 5 min after left mainstem bronchial occlusion

			HR, beats/min		MAP, mmHg		PAP, mmHg		Q_LPA_, µl⋅min^−1^⋅g^−1^		LPVRI, mmHg⋅min⋅g⋅ml^−1^	
Genotype	Treatment	*n*	Baseline	LMBO	Baseline	LMBO	Baseline	LMBO	Baseline	LMBO	Baseline	LMBO
*Ndufs4*^+/+^	Saline	13	566 ± 22	543 ± 25	78 ± 4	77 ± 4	17 ± 1	18 ± 1	104 ± 5	38 ± 2^a^	89 ± 2	219 ± 7^a^
*Ndufs4*^−/−^	Saline	9	566 ± 19	544 ± 21	80 ± 4	75 ± 3	19 ± 2	21 ± 2^a^	98 ± 8	59 ± 6^a,^^d^	99 ± 7	170 ± 13^a^
*Ndufs4*^+/+^	DMSO 100% (0.5 ml/kg)	11	573 ± 30	548 ± 36	85 ± 5	81 ± 5	16 ± 1	18 ± 2	99 ± 7	35 ± 4^a,^^c^	100 ± 7	243 ± 18^a^
*Ndufs4*^+/+^	Piericidin A (0.5 mg/kg)	7	571 ± 22	540 ± 28	66 ± 6^d^	70 ± 5	18 ± 1	18 ± 1	112 ± 8	77 ± 7^a,^^b,^^c,^^d^	76 ± 6	120 ± 14^a,^^b,^^d^
*Ndufs4*^+/+^	After 3 wk at ${\text{F}i}_{{\text{O}}_{\text{2}}}$ 0.11	10	570 ± 16	554 ± 14	69 ± 6	72 ± 6	24 ± 1^b,^^c^	27 ± 1^b,^^c^	89 ± 3	40 ± 4^a,^^c^	145 ± 6^b,^^c^	321 ± 23^a,^^b,^^c^
*Ndufs4*^−/−^	After 3 wk at ${\text{F}i}_{{\text{O}}_{\text{2}}}$ 0.11	11	604 ± 12	590 ± 16	79 ± 4	80 ± 5	26 ± 1^b,^^c^	28 ± 2^b,^^c^	86 ± 4	38 ± 3^a,^^c^	138 ± 10^b^	301 ± 26^a,^^c^

Values are means ± SE. The effect of LMBO on each parameter was analyzed in each group by ANOVA or Kruskal-Wallis test with a post hoc correction for multiple comparisons. Group differences of the same parameter in the same treatment group were compared by a paired *t*-test. HR, heart rate; LMBO, left mainstem bronchial occlusion; LPVRI, left pulmonary vascular resistance index; MAP, mean arterial pressure; PAP, pulmonary arterial pressure; Q_LPA_, left pulmonary arterial blood flow.

a *P* < 0.05 vs. baseline value of the same parameter in the same treatment group;

b *P* < 0.05 vs. saline-treated *Ndufs4*^+/+^;

c *P* < 0.05 vs. saline-treated *Ndufs4*^−/−^;

d *P* < 0.05 vs. *Ndufs4*^+/+^ mice in the same treatment group.

**Table 2.** Lower thoracic aortic flow and total systemic vascular resistance index measurements before (baseline) and 5 min after LMBO

			Q_LTAF_, µl⋅min^−1^⋅g^−1^		TSVRI, mmHg⋅min⋅g⋅ml^−1^	
Genotype	Treatment	*n*	Baseline	LMBO	Baseline	LMBO
*Ndufs4*^+/+^	Saline	5	197 ± 13	197 ± 12	153 ± 11	161 ± 16
*Ndufs4*^−/−^	Saline	5	233 ± 11	230 ± 8	154 ± 12	143 ± 7
*Ndufs4*^+/+^	DMSO 100% (0.5 ml/kg)	5	224 ± 7	228 ± 8	142 ± 10	154 ± 22
*Ndufs4*^+/+^	Piericidin A (0.5 mg/kg)	5	212 ± 10	202 ± 11	140 ± 16	151 ± 24
*Ndufs4*^+/+^	After 3 wk at ${\text{F}i}_{{\text{O}}_{\text{2}}}$ 0.11	5	187 ± 20	185 ± 22	167 ± 26	168 ± 17
*Ndufs4*^−/−^	After 3 wk at ${\text{F}i}_{{\text{O}}_{\text{2}}}$ 0.11	5	218 ± 20	205 ± 18	147 ± 14	148 ± 8

Values are means ± SE. The effect of LMBO on each parameter was analyzed in each group by ANOVA with a post hoc correction for multiple comparisons. ${\text{F}i}_{{\text{O}}_{\text{2}}}$, fraction of inspired oxygen; LMBO, left mainstem bronchial occlusion; Q_LTAF_, lower thoracic aortic blood flow; TSVRI, total systemic vascular resistance index.

Piericidin A blocks mitochondrial electron transfer by potently inhibiting CI (10, 40). To investigate whether pharmacological inhibition of CI impairs HPV, we measured the effect of piericidin A on LMBO-induced changes in pulmonary vascular resistance. Mice treated with piericidin A (dissolved in DMSO) had a smaller increase in LPVRI in response to LMBO than *Ndufs4*^+/+^ mice treated with DMSO alone (%increase in LPVRI in piericidin A- vs. DMSO-treated *Ndufs4*^+/+^ mice: 49 ± 12 vs. 144 ± 11%, *P* < 0.05, Fig. 1*B*). Hemodynamic parameters, including HR and LPVRI, did not differ between piericidin A- and DMSO-treated mice before LMBO (Table 1). Mice treated with piericidin A had a lower MAP at baseline than *Ndufs4*^+/+^ mice treated with DMSO (MAP in piericidin A- vs. DMSO-treated *Ndufs4*^+/+^ mice: 66 ± 6 vs. 85 ± 5, *P* < 0.05, Table 1). When compared with DMSO-treated *Ndufs4*^+/+^ mice, piericidin A did not alter Q_LTAF_ or TSVRI in response to LMBO (Table 2). Taken together, these results indicate that acute pharmacological inhibition of CI, similar to genetic deficiency of *Ndufs4*, impairs LMBO-induced HPV. The LMBO-related changes in LPVRI caused by piericidin A are not a result of alterations in systemic vascular resistance.

#### Ndufs4 deficiency does not induce pulmonary inflammation.

**Fig. 2.**
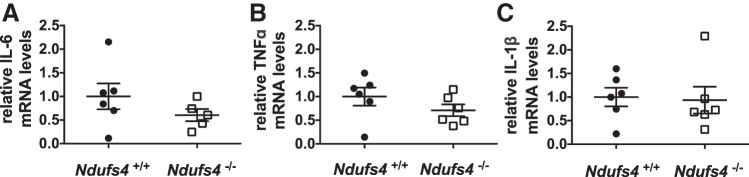
Relative levels of IL-6, TNFα, and IL-1β mRNA in lung tissue from air breathing *Ndufs4*^−/−^ and *Ndufs4^+/+^* mice. Levels of IL-6 (*A*), TNFα (*B*), and IL-1β (*C*) mRNA were similar between the two genotypes [*n* = 3 (male), *n* = 3 (female), *P* = not significant for each pair]. Group differences were compared by Student’s *t*-test. Data are expressed as means ± SE.

Previous studies showed that pulmonary inflammation may result in loss of HPV (25, 26, 38). To consider the possibility that Ndufs4 deficiency causes impaired HPV by inducing inflammation, we measured cytokine levels in lung tissue obtained from *Ndufs4*^−/−^ and *Ndufs4*^+/+^ mice breathing air. The levels of IL-6, TNFα, and IL-1β mRNA in the lung tissue of *Ndufs4*^−/−^ mice were not significantly increased compared with the levels of these cytokine mRNAs in the lungs of *Ndufs4*^+/+^ mice (Fig. 2, *A*–*C*).

#### Effect of chronic hypoxia on HPV and hemodynamic parameters in Ndufs4^−/−^ mice.

**Fig. 3.**
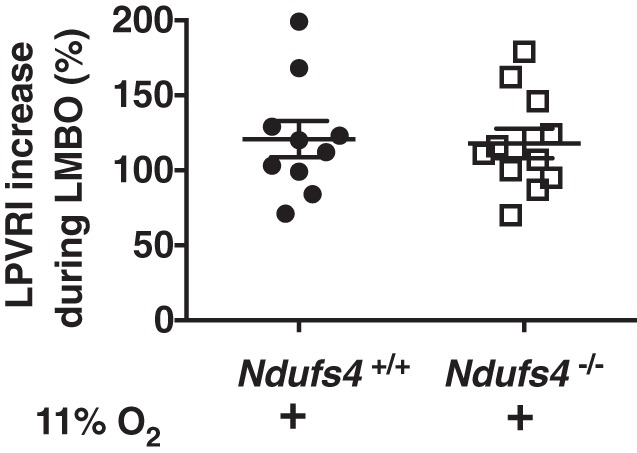
Hypoxic pulmonary vasoconstriction in Ndufs4*-*deficient mice breathing normobaric 11% O_2_ for 3 wk. The percentage increase in LPVRI in *Ndufs4*^−/−^ mice exposed to 11% O_2_ [*n* = 7 (male), *n* = 4 (female)] was comparable to that of 11% O_2_-breathing *Ndufs4^+/+^* mice [*n* = 7 (male), *n* = 3 (female), *Ndufs4*^−/−^ vs. *Ndufs4^+/+^*, *P* < 0.05]. LPVRI was calculated from the slope of the PAP/Q_LPA_ relationship at baseline and during LMBO. HPV is denoted as the percentage increase in LPVRI induced by LMBO. Groups were compared by Student’s *t*-test. Data are expressed as means ± SE. HPV, hypoxic pulmonary vasoconstriction; LMBO, left mainstem bronchial occlusion; LPVRI, left pulmonary vascular resistance index; PAP, pulmonary arterial pressure; Q_LPA_, left pulmonary arterial blood flow.

Continuous exposure of *Ndufs4*^−/−^ mice to normobaric 11% O_2_ prevents the development of neurological disease and markedly improves survival (9, 14). To investigate the effects of chronic hypoxia on LMBO-induced HPV in Ndufs4-deficient mice, *Ndufs4*^−/−^ and *Ndufs4*^+/+^ mice breathed 11% O_2_ for 3 wk. At baseline (before LMBO), breathing 11% O_2_ increased PAP and LPVRI in both *Ndufs4*^−/−^ and *Ndufs4*^+/+^ mice to a similar extent (Table 1). The increase in LPVRI induced by left bronchus occlusion was similar in *Ndufs4*^−/−^ and *Ndufs4*^+/+^ mice (%increase in LPVRI in *Ndufs4*^−/−^ and *Ndufs4*^+/+^ mice: 118 ± 10 vs. 121 ± 12%, *P* = not significant, Fig. 3). Hemodynamic parameters, including MAP and HR did not differ between the two groups either before or during LMBO (Table 1). To assess the effect of chronic hypoxia on systemic vascular resistance, we measured Q_LTAF_ in both genotypes. When compared with *Ndufs4*^+/+^ mice, breathing 11% O_2_ did not alter Q_LTAF_ or TSVRI in *Ndufs4*^−/−^ mice (Table 2). These results show that in Ndufs4-deficient mice the impairment of LMBO-induced HPV can be reversed by breathing 11% O_2_ for 3 wk and that there are no differences in systemic vascular resistance at baseline or during LMBO between chronically hypoxic Ndufs4-deficient and *Ndufs4*^+/+^ mice.

#### Effects of congenital Ndufs4 deficiency or pharmacological CI inhibition on arterial oxygenation and intrapulmonary shunt in air- or 11% O_2_-treated mice.

**Fig. 4.**
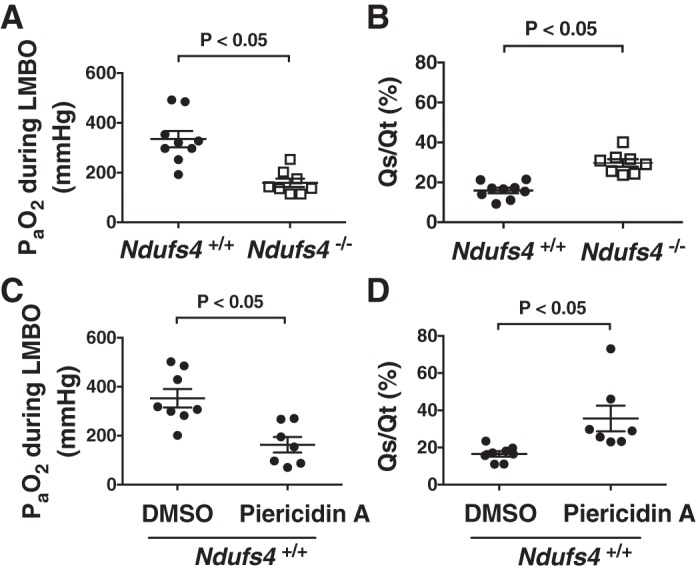
Arterial blood oxygen tension ($\text{P}{\text{a}}_{\text{O}}{}_{{}_{2}}$) and intrapulmonary shunt fraction (Qs/Qt) during LMBO in Ndufs4*-*deficient mice or *Ndufs4^+/+^* mice treated with complex I inhibitor piericidin A. During LMBO, the $\text{P}{\text{a}}_{\text{O}}{}_{{}_{2}}$ was lower in *Ndufs4*^−/−^ mice [*n* = 5 (male), *n* = 3 (female)], compared with *Ndufs4^+/+^* mice [*n* = 6 (male), *n* = 3 (female), *Ndufs4*^−/−^ vs. *Ndufs4^+/+^*, *P* < 0.05] (*A*). The calculated Qs/Qt fraction during LMBO was higher in *Ndufs4*^−/−^ mice [*n* = 4 (male), *n* = 4 (female)], compared with *Ndufs4^+/+^* mice [*n* = 5 (male), *n* = 4 (female), *Ndufs4*^−/−^ vs. *Ndufs4^+/+^*, *P* < 0.05] (*B*). After treatment of *Ndufs4^+/+^* mice with the complex I inhibitor piericidin A [*n* = 4 (male), *n* = 3 (female)], the $\text{P}{\text{a}}_{\text{O}}{}_{{}_{2}}$ was lower than in *Ndufs4^+/+^* mice treated with DMSO [*n* = 5 (male), *n* = 3 (female), piericidin A-treated *Ndufs4^+/+^* vs. DMSO-treated *Ndufs4^+/+^*, *P* < 0.05] (*C*). When compared with DMSO-treated *Ndufs4^+/+^* mice [*n* = 4 (male), *n* = 4 (female)], piericidin A [*n* = 4 (male), *n* = 3 (female)] treatment increased intrapulmonary shunt fraction (piericidin A-treated *Ndufs4^+/+^* vs. DMSO-treated *Ndufs4^+/+^*, *P* < 0.05) (*D*). Group differences were compared by Student’s *t*-test. Data are expressed as means ± SE. LMBO, left mainstem bronchial occlusion.

To assess the impact of impaired HPV on systemic oxygenation in *Ndufs4*^−/−^ and *Ndufs4*^+/+^ mice breathing air, arterial blood-gas analysis was performed during LMBO. In mice breathing air, the $\text{P}{\text{a}}_{\text{O}}{}_{{}_{2}}$ during LMBO was lower in *Ndufs4*^−/−^ than in *Ndufs4*^+/+^ mice ($\text{P}{\text{a}}_{\text{O}}{}_{{}_{2}}$ in *Ndufs4*^−/−^ vs. *Ndufs4*^+/+^ mice: 156 ± 20 vs. 335 ± 33 mmHg, *P* < 0.05; Fig. 4*A* and Table 3), consistent with impaired HPV in the former. To investigate whether pharmacological inhibition of CI also impairs systemic oxygenation during LMBO, arterial blood-gas analysis was performed in piericidin A-treated *Ndufs4*^+/+^ mice. When compared with DMSO-treated *Ndufs4*^+/+^ mice, piericidin A decreased arterial oxygenation during LMBO ($\text{P}{\text{a}}_{\text{O}}{}_{{}_{2}}$ in piericidin A- vs. DMSO-treated *Ndufs4*^+/+^ mice: 163 ± 32 vs. 352 ± 38 mmHg, *P* < 0.05; Fig. 4*C* and Table 3). An assessment of the intrapulmonary shunt (Qs/Qt) was used to investigate further the effect of disrupting normal CI function on the matching of pulmonary ventilation and perfusion. *Ndufs4*^−/−^ mice breathing air had a marked increase in Qs/Qt compared with *Ndufs4*^+/+^ mice (Qs/Qt in *Ndufs4*^−/−^ vs. *Ndufs4*^+/+^ mice: 26 ± 3 vs. 16 ± 1%, *P* < 0.05; Fig. 4*B*). Similarly, the Qs/Qt ratio in piericidin A-treated *Ndufs4*^+/+^ mice was greater than that in DMSO-treated control mice (Qs/Qt in piericidin A- vs. DMSO-treated *Ndufs4*^+/+^ mice: 36 ± 7 vs. 17 ± 1%, *P* < 0.05; Fig. 4*D*).

**Table 3.** Arterial blood gas analysis at the end of the hemodynamic studies in Ndufs4^−/−^ and Ndufs4^+/+^ mice breathing at 100% O_2_

Genotype	Treatment	*n*	pH_a_	$\text{P}{\text{a}}_{\text{O}}{}_{{}_{2}}$, mmHg	$\text{P}{\text{a}}_{\text{CO}}{}_{{}_{2}}$, mmHg	Hemoglobin, mg/dl	Base Excess, mmol/l
*Ndufs4*^+/+^	Saline	9	7.32 ± 0.02	335 ± 33	32 ± 3	10.8 ± 0.6	−8 ± 1.5
*Ndufs4*^−/−^	Saline	8	7.21 ± 0.03^b^	156 ± 20^b^	38 ± 3	11.6 ± 0.5	−11.3 ± 1.5
*Ndufs4*^+/+^	DMSO 100% (0.5 ml/kg)	8	7.32 ± 0.02	352 ± 38	34 ± 3	11.1 ± 0.3	−7.6 ± 1.6
*Ndufs4*^+/+^	Piericidin A (0.5 mg/kg)	7	7.28 ± 0.02	163 ± 32^a,^^b^	30 ± 1	10.6 ± 0.3	−11.6 ± 1.4
*Ndufs4*^+/+^	After 3 wk at ${\text{F}i}_{{\text{O}}_{\text{2}}}$ 0.11	7	7.22 ± 0.02^a^	269 ± 36	30 ± 2	16 ± 0.8^a^	−14.5 ± 1.1^a^
*Ndufs4*^−/−^	After 3 wk at ${\text{F}i}_{{\text{O}}_{\text{2}}}$ 0.11	7	7.14 ± 0.02^a^	336 ± 26	31 ± 1	15.1 ± 0.6^a^	−17.8 ± 1^a^

Values are means ± SE. Each parameter was analyzed in each group by ANOVA with a post hoc correction for multiple comparisons. ${\text{F}i}_{{\text{O}}_{\text{2}}}$, fraction of inspired oxygen; $\text{P}{\text{a}}_{\text{CO}}{}_{{}_{2}}$, partial pressure of arterial carbon dioxide; $\text{P}{\text{a}}_{\text{O}}{}_{{}_{2}}$, partial pressure of arterial oxygen.

a *P* < 0.05 vs. saline-treated *Ndufs4*^+/+^ mice;

b *P* < 0.05 vs. *Ndufs4*^+/+^ mice in the same treatment group.

**Fig. 5.**
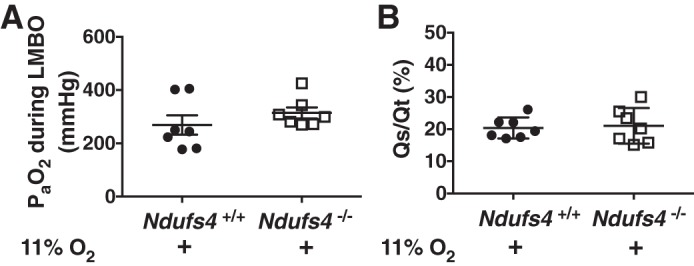
Arterial blood oxygen tension ($\text{P}{\text{a}}_{\text{O}}{}_{{}_{2}}$) and intrapulmonary shunt fraction (Qs/Qt) during LMBO in Ndufs4*-*deficient mice or *Ndufs4^+/+^* mice breathing 11% O_2_ for 3 wk. In *Ndufs4*^−/−^ mice, $\text{P}{\text{a}}_{\text{O}}{}_{{}_{2}}$ was increased after treatment with 11% O_2_ for 3 wk [*n* = 4 (male), *n* = 3 (female)] and was comparable to the level of $\text{P}{\text{a}}_{\text{O}}{}_{{}_{2}}$ in hypoxic *Ndufs4^+/+^* mice [*n* = 4 (male), *n* = 3 (female], *Ndufs4*^−/−^ vs. *Ndufs4^+/+^*, *P* = not significant) (*A*). The calculated Qs/Qt fraction was decreased in *Ndufs4*^−/−^ mice [*n* = 4 (male), *n* = 3 (female)] and comparable to the shunt fraction in *Ndufs4^+/+^* mice [*n* = 4 (male), *n* = 3 (female)] after breathing 11% O_2_ for 3 wk (*Ndufs4*^−/−^ vs. *Ndufs4^+/+^*, *P* = not significant) (*B*). Group differences were compared by Student’s *t*-test. Data are expressed as means ± SE. LMBO, left mainstem bronchial occlusion.

To characterize the effects of chronic hypoxia on systemic oxygenation in Ndufs4-deficient mice better, we also measured $\text{P}{\text{a}}_{\text{O}}{}_{{}_{2}}$ after breathing 11% O_2_ for 3 wk. *Ndufs4*^−/−^ mice exposed to 11% O_2_ showed a significant improvement in arterial oxygenation during LMBO compared with *Ndufs4*^−/−^ mice breathing air ($\text{P}{\text{a}}_{\text{O}}{}_{{}_{2}}$ in *Ndufs4*^−/−^ mice breathing 11% O_2_ vs. *Ndufs4*^−/−^ mice breathing air: 336 ± 26 vs. 156 ± 20 mmHg, *P* < 0.05, Table 3). After chronic exposure to 11% O_2_, arterial $\text{P}{\text{a}}_{\text{O}}{}_{{}_{2}}$ in *Ndufs4*^−/−^ mice was similar to that in *Ndufs4*^+/+^ mice breathing 11% O_2_ ($\text{P}{\text{a}}_{\text{O}}{}_{{}_{2}}$ in *Ndufs4*^−/−^ vs. *Ndufs4*^+/+^ mice breathing 11% O_2_: 336 ± 26 vs. 269 ± 36 mmHg, *P* = not significant; Fig. 5*A* and Table 3). Ndufs4-deficient mice breathing 11% O_2_ had a marked decrease in the intrapulmonary shunt (Qs/Qt) compared with *Ndufs4*^−/−^ mice breathing air. Qs/Qt ratio during LMBO was reduced to the levels of *Ndufs4*^+/+^ mice breathing 11% O_2_ (Qs/Qt in *Ndufs4*^−/−^ vs. *Ndufs4*^+/+^ mice breathing 11% O_2_: 22 ± 2 vs. 20 ± 1%, *P* = not significant; Fig. 5*B*). Taken together, these results show that intact CI function is required to maintain systemic oxygenation and physiological intrapulmonary Qs/Qt ratio during LMBO and that prolonged breathing of 11% O_2_ can improve arterial oxygenation in Ndufs4*-*deficient mice during LMBO.

#### Effect of chronic hypoxia on lung succinate and fumarate levels in Ndufs4^−/−^ and Ndufs4^+/+^ mice.

**Fig. 6.**
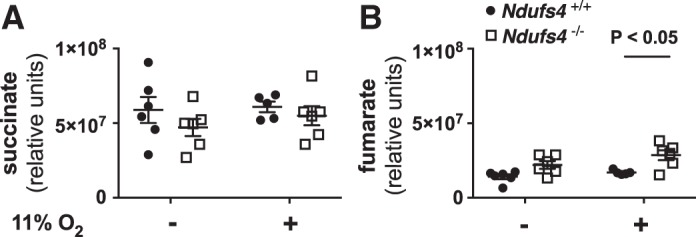
Lung tissue succinate and fumarate levels in *Ndufs4*^−/−^ and *Ndufs4^+/+^* mice exposed either to air or 11% O_2_. Succinate levels did not differ between the two groups either under normoxic or hypoxic conditions (*Ndufs4*^−/−^ vs. *Ndufs4^+/+^*, *P* = not significant) (*A*). *Ndufs4*^−/−^ mice breathing 11% O_2_ [*n* = 3 (male), *n* = 3 (female)] had higher fumarate levels compared with 11% O_2_ breathing control mice [*n* = 3 (male), *n* = 2 (female)], indicating increased oxidation of succinate and increased production of fumarate by CII (*Ndufs4*^−/−^ vs. *Ndufs4^+/+^*, *P* < 0.05) (*B*). Group differences were compared by ANOVA with a post hoc correction for multiple comparisons. Data are expressed as means ± SE. CII, mitochondrial complex II.

Changes in the function of CI have an impact on the overall activity of the ETC (11). Mitochondrial complex II (CII) is able to pass electrons, released by the oxidation of succinate, to coenzyme Q of the ETC thereby bypassing CI (4). To characterize better whether changes in the function of CII, induced by dysfunction of CI, might explain the observed improvement of HPV in *Ndufs4*^−/−^ mice breathing 11% O_2_, we measured whole lung levels of succinate and fumarate in air- and 11% O_2_-breathing *Ndufs4*^−/−^ mice. Succinate levels did not differ between the two groups either during normoxia or during hypoxia (Fig. 6*A*). *Ndufs4*^−/−^ mice breathing 11% O_2_ had higher fumarate levels compared with 11% O_2_-breathing control mice, consistent with increased oxidation of succinate and increased production of fumarate by CII (Fig. 6*B*).

#### Effect of chronic hypoxia on lung NADH/NAD^+^ ratio in Ndufs4^−/−^ and Ndufs4^+/+^ mice.

**Fig. 7.**
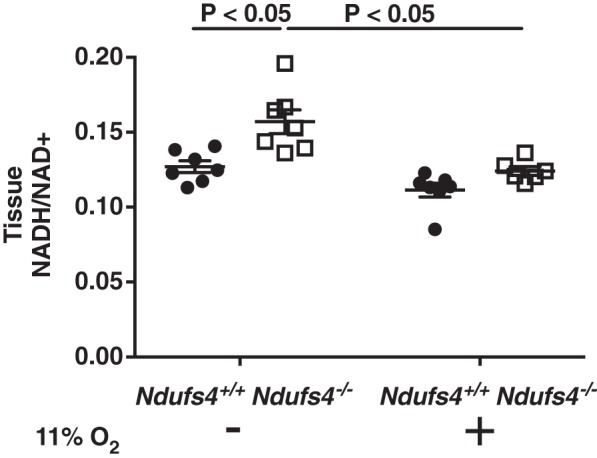
The ratio of NADH/NAD^+^ in the lungs of *Ndufs4*^−/−^ and *Ndufs4^+/+^* mice exposed either to air or 11% O_2_. The lungs of *Ndufs4*^−/−^ mice breathing air [*n* = 4 (male), *n* = 3 (female)] had an increased ratio of NADH/NAD^+^ compared with the lungs of *Ndufs4^+/+^* mice [*n* = 4 (male), *n* = 3 (female), *Ndufs4*^−/−^ vs. *Ndufs4^+/+^*, *P* < 0.05]. Breathing 11% O_2_ for 3 wk decreased the NADH/NAD^+^ ratio in *Ndufs4*^−/−^ mice [*n* = 3 (male), *n* = 3 (female)] compared with *Ndufs4*^−/−^ mice breathing air (*Ndufs4*^−/−^ breathing 11% O_2_ vs. *Ndufs4*^−/−^ breathing air, *P* < 0.05). Group differences were compared by ANOVA with a post hoc correction for multiple comparisons. Data are expressed as means ± SE.

In addition to maintaining the normal cellular energy state, mitochondria are essential for regulating cellular redox balance (32). To investigate whether alterations in redox balance might contribute to the loss of HPV, we measured NADH and NAD^+^ levels and calculated the ratio of NADH/NAD^+^ in lungs obtained from *Ndufs4*^−/−^ and *Ndufs4*^+/+^ mice breathing either air or 11% O_2_ for 3 wk. In mice breathing air, the lungs of *Ndufs4*^−/−^ mice had a higher NADH/NAD^+^ ratio than the lungs of *Ndufs4*^+/+^ mice (whole lung NADH/NAD^+^ ratio in *Ndufs4*^−/−^ vs. *Ndufs4*^+/+^ mice breathing air: 0.160 ± 0.008 vs. 0.126 ± 0.004, *P* < 0.05; Fig. 7). Breathing 11% O_2_ for 3 wk decreased the ratio of NADH/NAD^+^ in the lungs of Ndufs4-deficient mice but had no effect on the ratio of NADH/NAD^+^ in the lungs of *Ndufs4*^+/+^ mice (whole lung NADH/NAD^+^ ratio in *Ndufs4*^−/−^ vs. *Ndufs4*^+/+^ mice breathing 11% O_2_: 0.111 ± 0.005 vs. 0.124 ± 0.003, *P* = not significant; Fig. 7). Taken together, the results show that the lack of functional CI increases the ratio of NADH to NAD^+^ and chronic hypoxia (breathing 11% O_2_) improves redox homeostasis.

#### Effect of angiotensin II on constriction of the pulmonary vasculature in Ndufs4^−/−^ mice.

**Fig. 8.**
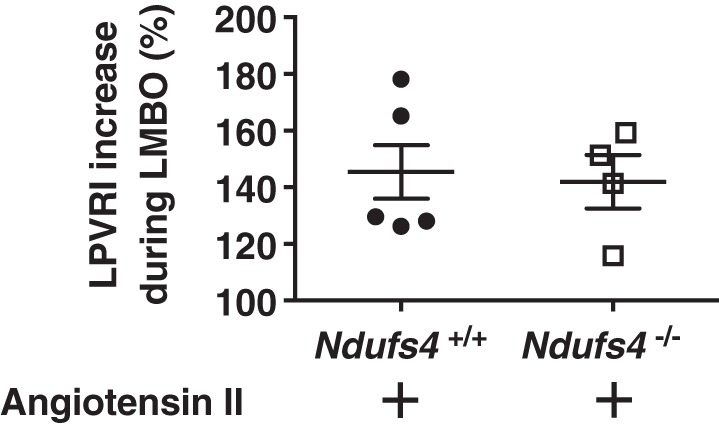
Left pulmonary vasoconstriction in angiotensin II-treated Ndufs4-deficient mice breathing air. The percentage increase in LPVRI in angiotensin II-treated *Ndufs4*^−/−^ mice [*n* = 2 (male), *n* = 2 (female)] was comparable to that of *Ndufs4^+/+^* mice [*n* = 4 (male), *n* = 1 (female), *Ndufs4*^−/−^ vs. *Ndufs4^+/+^*, *P* = not significant]. Group differences were compared by Student’s *t*-test. Data are expressed as means ± SE. LMBO, left mainstem bronchial occlusion; LPVRI, left pulmonary vascular resistance index.

To learn whether impairment of LMBO-induced HPV in *Ndufs4*^−/−^ mice is caused by nonspecific dysfunction of the pulmonary contractile apparatus, *Ndufs4*^−/−^ and *Ndufs4*^+/+^ mice were treated with intravenous angiotensin II. In *Ndufs4*^−/−^ mice, LPVRI increased from 107 ± 23 to 259 ± 57 mmHg·min·g·ml^−1^ (Table 4) during continuous infusion of angiotensin II (5 μg·kg^−1^·min^−1^). *Ndufs4*^+/+^ mice showed a similar increase in LPVRI from 88 ± 5 at baseline to 216 ± 12 mmHg·min·g·ml^−1^ during infusion of angiotensin II (%increase in LPVRI in angiotensin II-treated *Ndufs4*^−/−^ vs. *Ndufs4*^+/+^ mice: 142 ± 9 versus 145 ± 11%, *P* = not significant, Fig. 8). These results demonstrate that impairment of LMBO-induced HPV in *Ndufs4*^−/−^ mice is not a result of nonspecific dysfunction of the pulmonary vascular contractile apparatus.

**Table 4.** Hemodynamic measurements before (baseline) and 5 min after the start of angiotensin II infusion in Ndufs4^−/−^ and Ndufs4^+/+^ mice breathing air

				HR, beats/min		MAP, mmHg		PAP, mmHg		Q_LPA_, µl⋅min^−1^⋅g^−1^		LPVRI, mmHg⋅min⋅g⋅ml^−1^	
Genotype	Treatment		*n*	Baseline	ANG II	Baseline	ANG II	Baseline	ANG II	Baseline	ANG II	Baseline	ANG II
*Ndufs4*^+/+^	ANG II (0.5 µg·kg^−1^·min^−1^)		5	542 ± 36	620 ± 23^a^	82 ± 8	134 ± 5^a^	18 ± 1	26 ± 2^a^	88 ± 6	67 ± 6^a^	88 ± 5	216 ± 12^a^
*Ndufs4*^−/−^	ANG II (0.5 µg·kg^−1^·min^−1^)		4	506 ± 23	617 ± 9^a^	74 ± 5	138 ± 8^a^	19 ± 2	29 ± 3^a^	104 ± 17	61 ± 9^a^	107 ± 23	259 ± 57^a^

Values are means ± SE. Group differences were compared by Student’s *t*-test. The effect of angiotensin II (ANG II) on each parameter was analyzed in each group by a paired Student’s *t*-test. HR, heart rate; LPVRI, left pulmonary vascular resistance index; MAP, mean arterial pressure; PAP, pulmonary arterial pressure; Q_LPA_, left pulmonary arterial blood flow.

a *P* < 0.05 vs. baseline value of the same parameter in the same treatment group.

## DISCUSSION

The objective of this study was to investigate the role of CI in the regulation of HPV. Mice lacking the *Ndufs4* gene (*Ndufs4*^−/−^) and *Ndufs4*^+/+^ mice with pharmacological inhibition of CI had impaired HPV, which resulted in impairment of systemic oxygenation. The levels of cytokines in lung tissue obtained from *Ndufs4*^−/−^ mice breathing air were not significantly different from those in control mice, suggesting that impairment of HPV was not a result of pulmonary inflammation. Intravenous administration of angiotensin II to Ndufs4*-*deficient mice increased pulmonary resistance, showing that impairment of HPV was not a result of a dysfunctional pulmonary vascular contractile apparatus. In *Ndufs4*^−/−^ mice, breathing 11% O_2_ for 3 wk restored HPV and improved systemic oxygenation during LMBO. When compared with *Ndufs4*^+/+^ mice, *Ndufs4*^−/−^ mice breathing air had an increased whole lung ratio of NADH to NAD^+^. Restoration of HPV by breathing 11% O_2_ for 3 wk was associated with a normalized NADH/NAD^+^ ratio.

Children with Leigh syndrome usually die within the first years of life as a consequence of pneumonia and respiratory failure (8, 17). To our knowledge, no previous studies in humans or mice have examined whether HPV is impaired in states of a congenital complex I deficiency. If humans with Leigh syndrome have a similar defect in HPV as *Ndufs4*^−/−^ mice, then this defect may contribute to the high mortality of patients with Leigh syndrome.

The effects of complex I deficiency on systemic and pulmonary hemodynamics in *Ndufs4*^−/−^ mice has not been studied extensively. In the present study, we report that in young (35–40 days old) *Ndufs4*^−/−^ mice, complex I deficiency does not affect pulmonary hemodynamics under normoxic conditions. Previous studies showed that *Ndufs4*^−/−^ mice have altered cardiac function that progressively worsens with age (7, 28). However, in the present study, HR, blood pressure, and Q_LTAF_ did not differ between *Ndufs4*^−/−^ and *Ndufs4*^+/+^ mice before or during LMBO. The observed differences between the results in these studies may be attributable to the different ages of the mice that were studied and the different types of anesthesia used. Chouchani and colleagues (7) studied older mice (10–20 wk) and used isoflurane anesthesia to study cardiac function using MRI. In contrast, in this study we used younger mice (35–40 days old). In addition, because of concern that *Ndufs4*^−/−^ mice have increased sensitivity to inhaled anesthetics, we used a combination of fentanyl and ketamine while assessing cardiac function. Of note, Karamanlidis and colleagues (16) reported normal cardiac function at all ages in a murine model of cardiac-specific deletion of the *Ndufs4* gene.

As previously reported (9), *Ndufs4*^−/−^ mice had lower blood pH associated with lactic acidosis, compared with control mice. Complex I inhibition in *Ndufs4*^−/−^ mice leads to decreased oxidation of pyruvate inside mitochondria, resulting in increased pyruvate conversion to lactate with restoration of NAD^+^ needed for glycolysis. The effects of acidosis on HPV are incompletely understood; some studies showing increased HPV (3) whereas others reported that acidosis had no effect on HPV (24). It is possible that lower pH affected HPV in *Ndufs4*^−/−^ mice.

Previous investigators demonstrated that pharmacological inhibition of CI in isolated lung models impaired HPV, highlighting the importance of CI function in HPV (2, 23, 36, 37). Archer and colleagues reported in an isolated lung model that CI inhibition with rotenone, a second CI inhibitor, caused a transient increase in pulmonary perfusion pressure but abolished HPV when the lung was exposed to hypoxia. In the present study, we confirmed that pharmacological inhibition of CI, using piercidin A, impaired HPV. We did not observe an increase in baseline pulmonary vascular resistance in mice treated with piercidin A in vivo.

Archer and colleagues proposed that the redox state of the ETC acts as a sensor for oxygen and that changes in redox balance of the ETC reflect changes in alveolar oxygen levels (2, 29). In addition, changes in redox state may affect K^+^ channel gating and calcium signaling, which may alter pulmonary vascular smooth muscle contraction (1). Inactivation of the *Ndufs4* gene in mice has been reported to alter cardiac redox balance (16, 20). To examine whether redox imbalance contributed to the loss of HPV in *Ndufs4*^−/−^ mice, the levels of NADH and NAD^+^ in the lungs of Ndufs4-deficient mice were measured. Whole lung NADH was significantly increased in the lungs of air-breathing *Ndufs4*^−/−^ mice and was accompanied by a decrease in NAD^+^ levels, resulting in an increase in the NADH/NAD^+^ ratio compared with *Ndufs4*^+/+^ controls. The observed increase in whole lung NADH/NAD^+^ ratio may indicate alteration of the redox balance in *Ndufs4*^−/−^ mice breathing air that lead to the impairment of HPV.

Mitochondrial dysfunction in *Ndufs4*^−/−^ mice was previously reported by our group and other investigators (9, 14, 18, 27, 28). Calvaruso and colleagues (6) measured the complex I activity in multiple organs of *Ndufs4*^−/−^ mice and observed the lowest activity in lung tissue (9% of the control mice), compared with other organs, including brain, liver, and heart. Although measures of oxidative stress in the lung were not performed in the current study, previous investigators showed increased oxidative damage in the brain and fibroblasts of Ndufs4-deficient mice (15, 27, 34). Reactive oxygen species release has also been reported in the muscle and skin fibroblasts of *Ndufs4*-deficient patients (35). The level of superoxide production by fibroblasts from *Ndufs4*-deficient patients was inversely related to CI activity (35). Increased levels of reactive oxygen species in pulmonary smooth muscle cells may alter the function of potassium channels and thereby impair a signaling mechanism that is important for HPV.

We previously reported that hypoxia improves survival and reverses neurodegenerative disease in *Ndufs4*^−/−^ mice (9, 14). In these earlier studies, circulating lactate and α-hydroxybutyrate, both markers of tissue NADH/NAD^+^ ratios, increased with worsening disease and decreased with chronic breathing of 11% O_2_. In this study, breathing 11% O_2_ for 3 wk was found to restore HPV in *Ndufs4*^−/−^ mice and normalized the lung NADH/NAD^+^ ratio. The enhancement of HPV in *Ndufs4*^−/−^ mice breathing 11% O_2_ was associated with increased $\text{P}{\text{a}}_{\text{O}}{}_{{}_{2}}$ and reduced shunt fraction during LMBO.

Kruse and colleagues reported that CII activity was increased in the liver and skeletal muscle of *Ndufs4*^−/−^ mice (18). Supplemental succinate was also shown to reverse the effect of pharmacological CI inhibition and restore HPV (19). In the present study, the fumarate levels of the whole lung were higher in 11% O_2_ breathing *Ndufs4*^−/−^ mice compared with the controls, suggesting an increase in succinate oxidation as a possible compensatory mechanism in the setting of dysfunctional CI.

In conclusion, in the present study we demonstrated that mice congenitally lacking CI subunit Ndufs4 have impaired HPV and decreased systemic arterial oxygenation during LMBO. Similar results were found when CI was pharmacologically inhibited using piericidin A. The impaired HPV associated with Ndufs4 deficiency was not accompanied by either pulmonary inflammation or nonspecific vasomotor dysfunction. Breathing 11% O_2_ for 3 wk restored HPV in *Ndufs4*^−/−^ mice and improved systemic arterial oxygenation during LMBO. Restoration of HPV was accompanied by normalization of the whole lung NADH/NAD^+^ ratio. The murine results predict that if patients with Leigh syndrome have impaired HPV, they may be at risk to develop systemic hypoxemia because of ventilation/perfusion mismatch during episodes of pneumonia and respiratory failure. Our work motivates future investigations in patients with inherited mitochondrial disease to evaluate whether HPV is impaired and to what extent it may contribute to pathology in these patients.

## GRANTS

This work was supported by funds of the Marriott Foundation (to V. K. Mootha) and Department of Anesthesia, Critical Care and Pain Medicine at Massachusetts General Hospital (to W. M. Zapol) and by funds of the German Research Foundation (Deutsche Forschungsgemeinschaft WE 5471/2-1 and Cooperative Research Center 1149 to M. Wepler). V. K. Mootha is an Investigator of the Howard Hughes Medical Institute.

## DISCLOSURES

V. K. Mootha and W. M. Zapol are coinventors on a patent application submitted by Massachusetts General Hospital on the use of hypoxia as a therapy. V. K. Mootha owns equity stake in Raze Therapeutics and is a paid consultant for Janssen Pharmaceutics and 5AM Ventures.

## AUTHOR CONTRIBUTIONS

W.M.Z. conceived and designed of research; G.S., E.M., M.F., R.S., O.S., O.G., R.M.H.G., and K.P. performed experiments; G.S., E.M., M.F., R.S., O.S., R.M.H.G., K.P., M.W., and F.I. analyzed data; G.S., E.M., M.F., R.S., O.S., K.P., R.M., M.W., F.I., D.B.B., V.K.M., and W.M.Z. interpreted results of experiments; G.S., E.M., R.S., O.S., K.P., F.I., and W.M.Z. prepared figures; G.S., E.M., M.F., R.S., O.S., O.G., R.M.H.G., K.P., R.M., M.W., F.I., D.B.B., V.K.M., and W.M.Z. drafted manuscript; G.S., E.M., M.F., R.S., O.S., O.G., R.M.H.G., K.P., R.M., M.W., F.I., D.B.B., V.K.M., and W.M.Z. edited and revised manuscript; G.S., E.M., M.F., R.S., O.S., O.G., R.M.H.G., K.P., R.M., M.W., F.I., D.B.B., V.K.M., and W.M.Z. approved final version of manuscript.
